# Increasing Older Adults’ Social Connectedness: Development and Implementation of a Web-Assisted Acceptance and Commitment Therapy–Based Intervention

**DOI:** 10.2196/47943

**Published:** 2024-04-22

**Authors:** Amie Zarling, Joseph Kim, Daniel Russell, Carolyn Cutrona

**Affiliations:** 1 Department of Human Development and Family Studies, Iowa State University, Ames, IA, United States

**Keywords:** acceptance and commitment therapy, psychotherapy, loneliness, technology, lonely, older adults, older adult, gerontology, geriatric, geriatrics, emotion regulation, mental health, elder, elderly, isolation, aging, mHealth, digital health, digital mental health, online health, online support, eHealth, internet, depression

## Abstract

In this article, we will provide a rationale for a web-assisted acceptance and commitment therapy (ACT) approach to loneliness among older adults, drawing upon theories from the literature on adult development and aging, emotion regulation, and loneliness. The intervention program was developed using the principles of ACT, which is a cognitive behavioral approach and unified model of human behavior change and psychological growth. The ACT intervention focuses on developing nonjudgmental present-focused awareness of internal experiences (thoughts, emotions, and memories) through strategies such as acceptance and mindfulness rather than directly modifying or removing them per se. The ACT intervention appears well-suited to assist older adults in coping with the challenges of aging, as the focus is on an individual’s willingness to sit with internal experiences out of one’s control (ie, acceptance), stepping back from negative or critical thoughts and developing greater kindness toward oneself (ie, defusion), discerning what is most important to one’s true self (ie, values), and building larger patterns of effective action based on such values (ie, committed action). The ACT intervention was developed as a resource for older adults who are socially isolated or having difficulty with social connectedness. Eight modules comprise the web-assisted ACT intervention program, which includes reading materials, video clips, and activities. Each module is followed by a summary, a homework assignment, a short quiz to assess learning, and a moderated discussion with a coach. The intervention program begins with reconnecting participants with their values. The goal of the ACT intervention program is to foster flexibility in a participant’s behavior so they can behave consistently with their chosen values, rather than becoming locked into a pattern of behavior that is driven by avoiding distress or discomfort. The ACT intervention approach is both novel and innovative, as it is based on ACT and leverages a behavioral health web platform that is flexible and inclusive in its design. The ACT intervention aims to help older adults become more socially connected, less lonely, and more satisfied with their relationships with other people. The emphasis that ACT places on values and living life in accordance with one’s values renders it an approach ideally suited to older adults. Finally, recommendations for future research regarding this approach to addressing loneliness among older adults is addressed.

## Introduction

Loneliness, the subjective experience of feeling socially isolated, occurs at all stages of the life span [1]. Loneliness has been observed to impact older adults’ experience of depressive symptoms and lead to functional limitations [2,3]. In addition, loneliness has been found to impact older adults’ cognition, with loneliness positively associated with dementia [4-6]. Individuals who score high on loneliness visit physicians more frequently [7]. Loneliness has also been associated with cardiovascular risk and risk of mortality [3,8-10]. The crucial point from these studies is that across the life span, loneliness or lack of social relationships puts individuals at greater likelihood of mortality [11].

Older adults are an underserved population; the majority of older adults in need of mental health treatment do not receive care [12]. During the COVID-19 outbreak in early 2020, older adults were disproportionately affected with high rates of mortality and difficulty accessing telemedicine services [13]. COVID-19 has increased calls for the adoption of technology-based approaches to ensure older adults are able to access mental health services [14]. If older adults do receive treatment, it is typically pharmacological, despite the fact that many prefer counseling [15]. Reasons why older adults do not receive treatment include lack of knowledge about mental health services, lack of perceived need for services, and stigma [16,17]. In addition, provider factors such as biases and misconceptions about aging also contribute to mental health services being underused by older adults [12,18].

To date, most research examining the effectiveness of psychosocial treatments with older adults has examined cognitive behavioral approaches. Studies have shown that these types of treatments are effective in treating depression in older adults in the community and primary care [19]. However, studies have produced mixed findings on their effectiveness with some subpopulations, such as depressed, medically ill, homebound older adults [20,21]. Cognitive behavioral therapy (CBT) approaches may also be suboptimal in the treatment of anxiety in later life [22,23]. Thus, there is a need to further develop and test psychosocial treatments to provide better care to older adults.

The purpose of this paper is to present an acceptance and commitment therapy (ACT)–based intervention program comprising a cognitive-behavioral web-assisted intervention to enhance social connectedness in older adults. We will review the literature on evidence-based programs for older adults and provide the theoretical basis for our ACT-based intervention program [24]. We will review the rationale for such an approach and provide a brief description of the ACT intervention program, as well as describe current evaluation efforts and future directions for the work.

## Ethical Considerations

The study was approved by the New England Institutional Review Board (IRB tracking number: 120180244).

## Loneliness Interventions

The deleterious impact of loneliness on physical and mental health points to the need for evidence-based intervention programs for older adults. Few evidence-based interventions have been developed for older adults, and creative approaches are needed. There have been various intervention approaches for addressing loneliness. Interventions approached at the individual level have included CBT, interpersonal therapies, and psychoanalytic therapies; interventions at the group level have focused on social skills training and groups centered around addressing shyness and depressive symptoms [25]. For older adults, interventions that may lessen the experience of loneliness have focused on social resources (eg, family and friends) and individual pursuits (eg, spending time on meaningful activities) [26].

Meta-analyses on the effectiveness of loneliness interventions have pointed to the effectiveness of psychological interventions [27,28]. A recent meta-analysis was conducted to specifically examine the effectiveness of psychological interventions in reducing loneliness [27]. The effectiveness of the various types of interventions did not significantly differ; however, reminiscence therapy had the largest effect size, followed by social identity interventions; CBT had the lowest effect size [27]. Similar results were observed in another meta-analysis where reminiscence therapy and social skills training had the largest effect size, followed by CBT [28]. However, there was only 1 study that used reminiscence therapy. In addition, the meta-analysis found higher effect sizes for intervention studies that (1) used the UCLA Loneliness Scale to assess loneliness, (2) used technology (telephone or computer) to deliver the intervention, and (3) addressed maladaptive social cognition [28]. This meta-analysis indicated that the use of technology (ie, telephone or computer) in delivering the intervention can be an effective way to address loneliness [28].

A major challenge in developing evidence-based programs for older adults has been access. Telehealth, the use of video conferencing to provide social support for older adults, is one approach that has been used during COVID-19 [29]. Interventions delivered through technology (ie, telephone or computer) have been found to be effective at reducing loneliness for older adults. One study examined the effectiveness of delivering a web-assisted intervention to older adults with chronic illness or handicap and found that computer and internet use significantly reduced loneliness among older adults [30]. In addition, lower mean levels of loneliness and depressive symptoms were found in a videoconferencing program for older adults in nursing homes after administering the intervention for 3 months [31]. Educational programs aimed at teaching older adults to use computers and the internet have also been found to significantly reduce loneliness [32]. In addition, a recent randomized controlled trial during COVID-19 examining the effectiveness of a web-assisted group intervention via Zoom found that it was effective in reducing loneliness and depressive symptoms among older adults [32]. A qualitative study examining the impact of a web-assisted intervention on loneliness among adults aged 18 to 64 years found that technology was viewed as a means of social connection. Web-assisted interventions have the ability to have a positive impact, as reviewed above. They can present information in a simple format (eg, video, graphics, audio), reach people in their homes and in rural areas, and reduce stigma related to seeking treatment. However, too much use of technology can lead to videoconferencing fatigue, and face-to-face communication is preferred [33].

## Cognitive Approach to Loneliness

Theoretical perspectives on loneliness have encompassed a wide range of approaches. Weiss [34] presented an interactionist view of loneliness that focused on the types of relationship deficits in people’s social networks. He described 2 types of loneliness: emotional loneliness (absence of a close partner) and social loneliness (absence of friendships and community). Other scholars have advocated for a cognitive approach to loneliness, which emphasizes the discrepancy between individuals’ *subjective perceptions* of their social life and their *desired quantity or quality* of social relationships [35]. That is, loneliness is hypothesized to occur when individuals’ networks of social relationships do not meet their expectations [35]. Cognition can act as a mediator between perceived loneliness and the intensity of the experience. For example, loneliness is often increased or decreased based on one’s thoughts and beliefs about one’s social skills. If a person believes that they are awkward and drive other people away, they may view every failed social encounter as their fault and as something that cannot be improved in the future. Thus, the cognitive approach emphasizes causal attributions for social difficulties, as well as behavioral and personality traits.

Based on the cognitive theory of loneliness and past research examining the effectiveness of psychological interventions, a new approach to loneliness is warranted that uses technology to deliver the intervention components. The use of technology (ie, telephone or computer) has been observed to be an effective means of delivering an intervention [27,28]. Psychosocial interventions (ie, reminiscence therapy, social identity interventions, and CBT) have been observed to be effective in reducing the experience of loneliness [27,28]. A systematic review of communication technology interventions suggested a need for further studies in the fields of loneliness and web-based technologies to identify opportunities to reduce loneliness in older people [36]. We propose that ACT, an emerging evidence-based approach to the treatment of emotional distress, is a viable new intervention approach for addressing loneliness among older adults, delivered via computer or the internet [24].

## ACT-Based Intervention

The intervention program was developed by drawing upon basic research on loneliness in older adults and using the principles of ACT. ACT is a cognitive-behavioral approach and unified model of human behavior change and psychological growth. ACT interventions focus on developing nonjudgmental present-focused awareness of internal experiences (thoughts, emotions, and memories); willingness to sit with internal experiences that are out of one’s control (ie, acceptance); stepping back from negative or critical thoughts and developing greater kindness toward oneself (ie, defusion); discerning what is most important to one’s true self (ie, values); and building larger patterns of effective action based on such values (ie, committed action). Compared to traditional CBT, ACT pays greater attention to the context and functions of private events and emphasizes helping individuals respond to them with greater flexibility through strategies such as acceptance and mindfulness rather than directly modifying or removing them per se.

There are several lines of evidence that support the use of ACT with older adults who are socially isolated or having difficulty with social connectedness. First, theories of adult development and successful aging suggest that an ACT approach to treatment could be useful for older adults [37]. It is well established that older adults experience a change in life dynamics due to shifts in gains and losses. Older adulthood has a higher proportion of losses and many of those are out of one’s control and unable to be changed [38]. Given that research has consistently shown that loneliness in older adults can be exacerbated by losses such as disability [39,40], decreased mobility [41-43], and widow- or widowerhood [43-45], it may prove fruitful to consider intervention approaches that assist older adults to respond flexibly to these relatively unmodifiable aspects of later life. The use of interventions was proposed to enhance psychological acceptance among older adults in response to findings of higher levels of well-being among older adults who showed greater psychological acceptance [46]. Also, in a study in which treatment response was defined as a reduction in the amount of interference with life due to pain, older people were more likely to respond to ACT compared to CBT [22].

Second, althought CBT approaches have been used with some success, there is reason to doubt their effectiveness for all individuals. Traditional CBT strategies that involve challenging the validity of thoughts may not be beneficial to older adults because the thoughts and feelings that arise after losses, while unhelpful, may not be unrealistic. Moreover, because older adults often have beliefs about aging that have solidified over a long period of time, modifying them may not be an efficient use of time [47].

This is consistent with research on CBT for mental health problems, which has revealed that the cognitive restructuring components of CBT do not significantly improve therapeutic outcomes [48]. Such techniques may be invalidating, futile, or cause iatrogenic effects. For example, there are data to show that cognitions are not directly modifiable and that deliberate attempts to change or suppress thoughts can increase their occurrence and behavioral impact [49]. Furthermore, some evidence suggests that older adults who make active efforts to eliminate problems that cannot be solved are at a higher risk for depression and other negative outcomes, and that disengaging from commitments or goals that are unattainable—followed by choosing an attainable alternative—is associated with better emotional well-being [50,51]. An acceptance approach in which individuals learn to focus on their remaining resources may be more beneficial than an approach in which they are encouraged to modify their thinking about loss or disability.

Third, the goal of an ACT intervention is to live life in accordance with deeply held values. ACT may be particularly appropriate for older adults because individuals experiencing declines associated with aging may limit their goals to those that are most highly valued, work harder to strive toward achieving those goals, and use alternative strategies to compensate for formerly used strategies that may no longer be workable. This perspective is articulated in the Selective Optimization with Compensation Model of successful aging [52]. This is the essence of ACT and psychological flexibility. Such a model of treatment may resonate with older adults, which in turn may make it more likely they will actively engage in treatment. For example, there is some evidence to suggest that attrition rates may be lower among older adults treated with ACT when compared to those who received CBT [22].

In sum, ACT appears well-suited to assist older adults in coping with the challenges of aging, such as losses in functioning and changes in social connections. ACT has been shown to be effective for older adults in other areas, such as reducing depression and anxiety and improving symptoms of chronic pain [53-55]. ACT interventions have been adapted for web-assisted delivery and have been shown to be effective in teaching ACT-based skills to manage conditions such as overweight, fibromyalgia, and stress [56-58]. Recent research indicates even 4 web sessions can have a positive impact [59].

## Development of a Web-Based ACT Intervention for Older Adults

Table 1 provides an overview of the ACT intervention program components. The ACT intervention program begins with reconnecting the client with their values, defined as intrinsic reinforcers, which provide a chosen direction for their behaviors and actions despite obstacles faced [60]. First, a list of relationships is provided (friendships, romantic or intimate relationships, family relationships, acquaintances or neighbors, and new people) and participants are asked to indicate how much they value each of those relationships. Importantly, indicating the level of importance means what the participants would like to see in their life, and does not necessarily mean that those relationships are currently matching up with that desired level. Next, a list of potential characteristics and qualities that people often value in their relationships is provided, and the client chooses the 5 that are most important to them. The list includes qualities such as being trustworthy, accepting, spontaneous, kind, open, sincere, forgiving, and loyal. The ultimate goal of the ACT intervention program is to foster flexibility in the participant’s behavior so they can behave consistently with their chosen values, rather than becoming locked into a pattern of behavior that is driven by avoiding distress or discomfort.

**Table 1.** Acceptance and commitment therapy intervention program modules.

Module	Purpose
(1) Introduction and values	Education about loneliness and why it is important to address loneliness Education about values Identifying and connecting with values and what the older person wants relationships to look like, to enhance motivation
(2) Exploring coping	Awareness of triggers and consequences Identifying how one copes with distress Evaluating the workability of one’s coping strategies
(3) Awareness of thoughts	Learning how the mind works and how thoughts influence behavior Learning how some people are often sensitive to certain social situations
(4) The impact of thoughts	Identifying one’s own sticky thoughts and how they keep one stuck
(5) Changing the impact of one’s thoughts	Stepping back from thoughts Labeling thoughts
(6) Changing the impact of ones’ thoughts	Acceptance Self-compassion
(7) Social stuck points	Identifying one’s interpersonal patterns and identifying where one might need skills Learning different skills for finding the relationships one wants
(8) Putting it all together	Integrating education and skills from modules 1-7 Identifying barriers and setbacks and making a plan for skill use
Homework	Self-assignments after each module on behavioral tasks to provide exposure to previously avoided situations, with the goal of increasing skill use and increasing adaptive thought patterns

This intervention program is an interactive, dynamic web-based intervention for social isolation and loneliness that delivers ACT over 8 interactive modules. The web-assisted modules are largely self-paced. The ACT intervention includes reading materials, video clips, and activities; each module is followed by a summary and homework assignment, a short quiz to assess learning, and a moderated discussion with a coach. Participants also have access to supplementary materials and email reminders. Participants are instructed to work through the modules in sequential order, approximately one module per week. Each module begins with a brief review of the content from the previous module before introducing a new concept. The homework includes self-assigned behavioral activities to complete each week (eg, “set a specific behavioral goal to engage in this week where you practice the skill learned in this week’s module”), and the participant is prompted to enter a description of the self-assignment after the module. Before starting the next module, participants are prompted to complete an “activity recap,” where they enter whether or not they completed the behavioral exercise, and to indicate their satisfaction with the exercise. Finally, they are asked to share any challenges that arose while engaging in the activity, and if this kept them from completing it. Participants are sent reminders if they have not logged on to the system for over a week.

The exercises in the first modules of the ACT intervention program attempt to increase clarity about what participants truly value in relationships and social connections, examine the ways in which current behaviors are either helping or hurting these values, and help participants begin to take concrete steps toward behaving consistently with their social values. This is important to set the stage for the rest of the program. All educational and skill-building activities in the ACT intervention program are linked to participants’ values, and the module content is aligned with living life in accordance with their own personally chosen values. For example, an increased willingness to experience one’s distressing thoughts and emotions (instead of trying to change them) is a skill that is explored as a way to facilitate progress in areas of one’s life that provide meaning and personal fulfillment. Participants’ self-identified values are also used to set values-consistent goals. For example, if they have an overall goal to have more friendships, their specific goal might be to participate in more social activities where they can meet new people. Most importantly, the homework assignments at the end of each module (ie, self-identified behavioral tasks) are based on what is personally meaningful for each participant. For example, one participant cited being open, friendly, and active as key values. Her homework assignment was to attend the community’s senior center game night and talk to a person she had not met previously. Fostering these patterns of committed action is highly important, as this is what helps the participant increase their ability to engage in behaviors consistent with their values and increase social connectedness.

Next, the participants reflect on their use of coping strategies (ie, avoidance and control-based strategies) and evaluate if these are helpful. The goal of this module is to help the participants recognize that avoidance often does not work, and in fact may be their primary problem. For example, isolating oneself at home to control the uncomfortable feelings that arise when attempting to meet new people is an avoidant and unhelpful coping strategy. To address maladaptive coping strategies, participants are guided through several activities to identify positive and active coping strategies. It is even better if their active coping strategies also involve building social connections.

The next several modules focus on thoughts, particularly how the mind works in social situations and how thoughts might influence participants’ behavior. These modules also emphasize how some people are vulnerable to experiencing certain thoughts more often, especially in social situations (eg, sensitivity to rejection). Participants identify “sticky thoughts,” which are thoughts that tend to stick around and control one’s behavior, even when they are unhelpful. For example, a sticky thought might be “Nobody wants to get to know me.” Participants are encouraged to increase awareness of how these thoughts get in the way of relationships with other people. Participants are then introduced to several strategies for changing the impact of their sticky thoughts, such as defusion (ie, stepping back from thoughts), acceptance, and self-compassion. One session is then devoted to recognizing social situations in which to use their newly learned skills (eg, when meeting someone new, making conversation with someone, or experiencing conflict with someone). In the final part of the program, participants reflect on their values and set further goals to live their lives according to these values, solving problems related to any perceived barriers.

Several adaptations were made to the program to ensure that it was appropriate for older adults. Changes in cognitive functioning with aging are not universal and older adults show extensive variability, but it is important to make programming flexible and inclusive for all learning abilities and types. Older adults may experience a decline in cognitive speed, working memory, selective attention, and fluid intelligence. Research indicates that for people with cognitive impairment, information should be presented slowly and with frequent repetitions and summaries.

In the ACT intervention program, information is presented in multiple ways, and participants are encouraged to notice what does and does not work for them. To accommodate for a decline in some aspects of executive functioning, such as memory, new information is presented in the context of the previous module’s material. Participants can revisit module content at any time, including readings, videos, and assignments. Phone prompts or alarms can remind participants in the program to carry out certain activities, such as behavioral homework tasks.

## Conclusion

The desire for fulfilling social connections is a universal need and essential to well-being. Involvement in satisfying social relationships contributes to enhanced emotional and physical health throughout the life span. The fulfillment of this need looks different for older adults, as they navigate the developmental changes of later life and adjust to gains and losses that occur in their social networks. Furthermore, factors such as fear of intimacy, low self-esteem, and behavioral struggles such as difficulty with social skills may exacerbate feelings of loneliness and also make it more difficult to recover from loneliness.

This intervention aims to help participants become more socially connected, less lonely, and more satisfied with their relationships with other people. Researchers and clinicians collaborated on the ACT intervention program, leveraging a behavioral health platform to deliver the intervention. Most existing web-assisted interventions for loneliness that have been tested for efficacy are therapist-based and require extensive involvement by mental health professionals, and they are therefore neither anonymous nor self-directed [27]. Unlike therapist-assisted programs on the web, the ACT intervention program is an interactive, dynamic web-based intervention for social isolation and loneliness that delivers material over 8 modules and can be done largely at one’s own pace and anonymously. A trained wellness coach is available to chat (voice or web-assisted) after each module to assist the user with processing program content or answering questions.

The ACT intervention approach is both novel and innovative, as it is based on ACT and leverages a behavioral health web-assisted platform that is flexible and inclusive in its design. The session content of the ACT intervention program is reflective of current knowledge of loneliness presented in the literature, as well as the extant evidence on ACT for older adults. The emphasis that ACT places on values and living life in accordance with one’s values renders it an approach ideally suited to older adults. Furthermore, some of the basic notions of ACT (eg, that pain and suffering are inevitable in human experience and that trying to avoid pain and suffering leads to problems) are highly compatible with this developmental stage of the life span and consistent with basic research on older adults and well-being.

Future studies should include randomized controlled trials of the ACT intervention program that include an attention control group, rater-blind assessments, and a systematic investigation of possible mechanisms of action (eg, acceptance). Measures of treatment adherence will need to be established, and interactions with coaches should be monitored and assessed for treatment fidelity and competency. Using mediator-moderator analyses, physical and cognitive functioning should be evaluated as moderators, and intrinsic motivation and homework adherence as mediators of the ACT intervention program’s effectiveness. Additional research may include determination of optimal “dosages” (frequency and duration) of the program for different subgroups of participants. Finally, an examination of treatment components via dismantling studies may reveal the program activities that are most potent in reducing loneliness. For example, the extent to which chat with coaches is essential to the program’s success will need to be studied.

## Abbreviations

ACT: acceptance and commitment therapy
CBT: cognitive behavioral therapy
